# Stage-dependent clinical burden in hospitalized Parkinson’s disease with a positive bedside orthostatic hypotension screen: a retrospective cross-sectional analysis

**DOI:** 10.3389/fnagi.2026.1894279

**Published:** 2026-08-12

**Authors:** Hengzhang Ma, Zhixiong Zheng, Caihua Chen, Mingyuan Xu, Qiaohui Zhu, Shenkun He

**Affiliations:** Department of Neurology, Mindong Hospital, Ningde, Fujian, China

**Keywords:** China, fractures, Hoehn–Yahr stage, hospitalization, orthostatic hypotension, Parkinson’s disease, sleep

## Abstract

**Background:**

Orthostatic hypotension (OH) is common in Parkinson’s disease (PD), but what is known of its clinical associations comes from ambulatory or population-based cohorts assessed under protocolised or tilt-table conditions. The patients whom a routine bedside screen identifies on a general ward have not been described.

**Objective:**

To characterise the modified Hoehn–Yahr (H–Y) stage distribution of hospitalized PD patients with a positive bedside OH screen, and to describe how clinical burden is distributed across stage within that group.

**Methods:**

Retrospective cross-sectional analysis of 125 consecutive adults admitted to a Chinese tertiary neurology department (January 2021 to March 2025) with idiopathic PD and a positive bedside OH screen, defined as a fall of ≥20 mmHg systolic or ≥10 mmHg diastolic at 1 or at 3 min upright. No OH-negative comparator was available. The primary outcome was the H–Y stage distribution; secondary outcomes were motor severity (MDS-UPDRS Part III), motor complications, fracture history, physician-documented sleep disturbance and length of stay. Stage groups were compared using rank-based tests with effect sizes, with multivariable and Firth penalized logistic regression for the two principal binary outcomes.

**Results:**

Screen-positive inpatients spanned the whole H–Y spectrum: 42.4% (95% CI 34.1–51.2) were at H–Y 1.0–2.0, before postural instability. One third (33.6%, 95% CI 25.9–42.3) met the criterion at 1 min only and would not have been detected by a 3-min-only rule. Motor severity rose steeply across stage (median MDS-UPDRS Part III 18, 35 and 62.5; *ε*^2^ = 0.65), as did motor complications (*ε*^2^ = 0.52), fracture history (5.3%, 37.9%, 30.0%) and length of stay (*ε*^2^ = 0.10). Physician-documented sleep disturbance instead peaked at the middle stage (30.3%, 58.6%, 45.0%), persisting after adjustment (odds ratio 2.44, 95% CI 1.01–5.92).

**Conclusion:**

A bedside OH screen identifies a clinically heterogeneous inpatient group that is not confined to advanced disease, and a third of it is detectable only at 1 min. Because all participants were screen-positive, these comparisons describe that group and cannot establish that the screening result marks burden independently of stage.

## 1 Introduction

Parkinson’s disease (PD) is the fastest-growing neurological disorder worldwide, and its burden is rising particularly steeply in China (Ben-Shlomo et al., 2024; Meng D. et al., 2024), where modelling of incidence and mortality projects continued growth through the end of this decade and a corresponding rise in the number of patients requiring inpatient care (Chen et al., 2022; Lan et al., 2024).

Cardiovascular autonomic dysfunction is among the most consequential non-motor features of PD (Xu et al., 2025; Peña-Zelayeta et al., 2025). Orthostatic hypotension (OH), defined by consensus as a fall of at least 20 mmHg systolic or 10 mmHg diastolic blood pressure within 3 min of standing or head-up tilt (Freeman et al., 2011), affects roughly a third of patients with PD: a recent meta-analysis of 7,748 participants estimated a pooled prevalence of 33.1% (95% CI 29.3–37.0) and found that patients with OH were older and had longer disease duration than those without (Wang et al., 2025; Velseboer et al., 2011). OH in PD is associated with impaired gait and balance (Imbalzano et al., 2024), falls (Zhang et al., 2024), cognitive decline (Hiorth et al., 2024; Longardner et al., 2020; Bagheri et al., 2026), reduced quality of life (Meng Y. et al., 2024) and greater healthcare utilization (Merola et al., 2018). The classic definition, however, is not the whole picture. Continuous blood-pressure monitoring shows that transient orthostatic hypotension, a fall that resolves within the first minute of standing, is at least as common in PD as the classic sustained form and is associated with orthostatic intolerance and syncope, yet is readily overlooked by intermittent bedside measurement (Fanciulli et al., 2020).

Almost everything known about the clinical correlates of OH in PD derives from ambulatory or population-based samples assessed under protocolised conditions or by tilt-table testing (Wang et al., 2025; Hiorth et al., 2024; Yoo et al., 2023). Hospitalized patients are a different population, in whom acute illness, deconditioning, immobility and polypharmacy converge, and it is on the ward rather than in the autonomic laboratory that a bedside blood-pressure check is actually performed. What is not known is who a routine bedside screen picks up. If it selects mainly patients with advanced disease, it adds little to a stage assessment already made at the bedside; if it selects across the spectrum, it identifies a group whose burden a clinician cannot infer from stage alone.

We therefore describe, in a Chinese tertiary neurology department, the modified Hoehn–Yahr (H–Y) stage distribution of PD inpatients identified by a routine bedside OH screen, and characterise how motor, non-motor and resource-use burden is distributed across stage within that group. The question addressed is not whether OH causes greater burden, which this design cannot answer, but what a clinician should expect when a ward screen is positive, and whether the answer depends on the patient’s stage.

## 2 Methods

### 2.1 Study design and setting

This was a retrospective cross-sectional analysis of a hospitalized cohort at the Department of Neurology of Mindong Hospital, a tertiary referral centre serving Ningde municipality in Fujian Province, southeastern China. All exposures and outcomes were ascertained during a single index admission and no participant was followed after discharge; we use “cross-sectional” for that reason, and reserve “cohort” for the group of patients rather than as a description of the design. Reporting follows the STROBE recommendations for cross-sectional studies (von Elm et al., 2007), with the completed checklist provided as Supplementary File 1. The study was approved by the Ethics Committee of Mindong Hospital and the requirement for individual consent was waived (Declarations).

### 2.2 Participants

Consecutive admissions between 1 January 2021 and 31 March 2025 with a primary discharge diagnosis of idiopathic PD (ICD-10 G20) were identified from the hospital’s clinical data warehouse. The diagnosis was made by the attending neurologist according to the Movement Disorder Society (MDS) clinical diagnostic criteria (Postuma et al., 2015); at data extraction every record was reviewed against those criteria and cases not supported by the documented findings were excluded.

**Figure 1:**
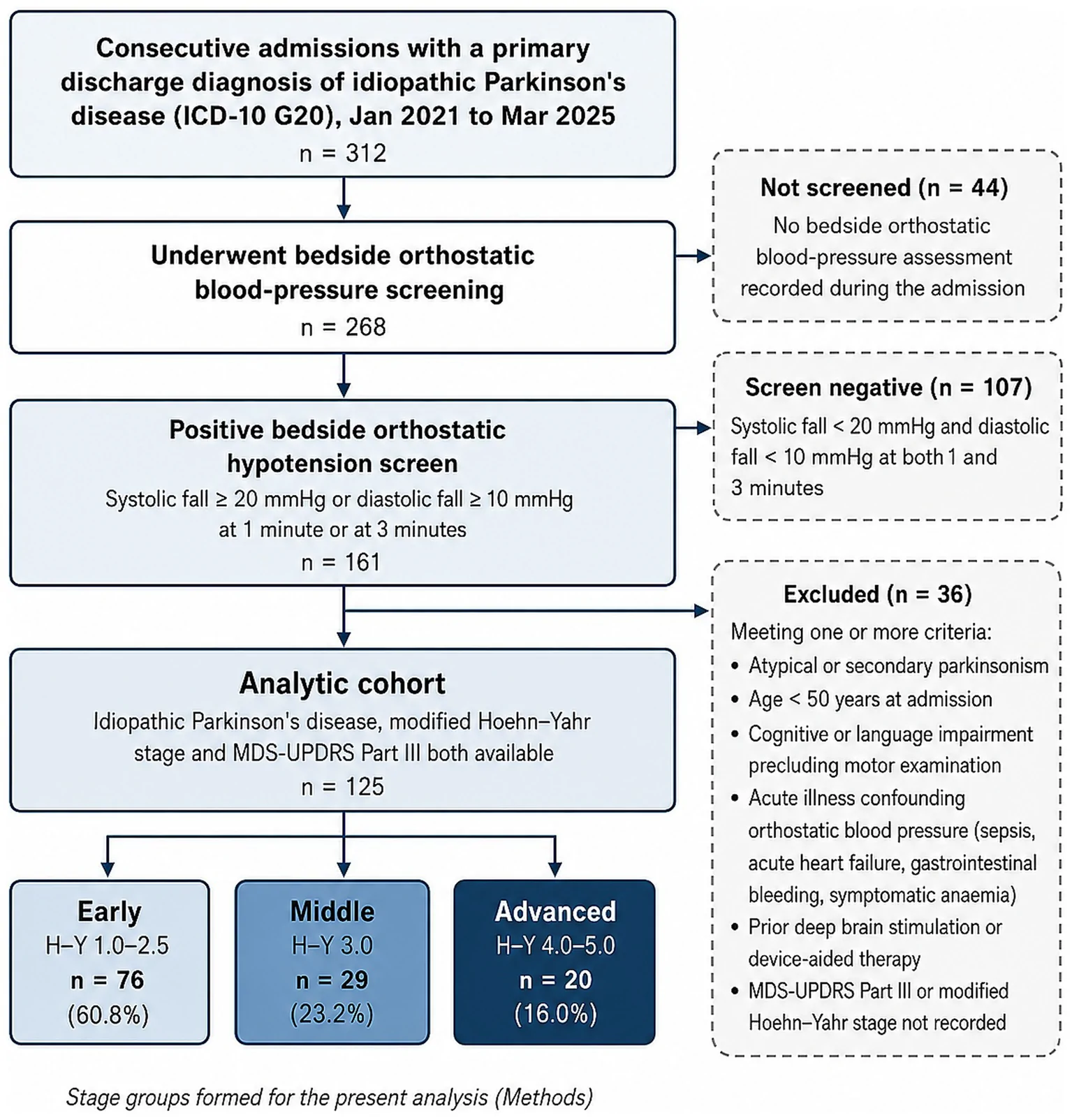
Participant flow. Consecutive admissions to the Department of Neurology of Mindong Hospital between 1 January 2021 and 31 March 2025 with a primary discharge diagnosis of idiopathic Parkinson’s disease (ICD-10 G20). A positive bedside screen required a fall of ≥20 mmHg systolic or ≥10 mmHg diastolic from the supine baseline at the 1-min or the 3-min reading; fulfilment at either time point was sufficient (Methods, section 2.3). Stage groups were formed for the present analysis on clinical grounds and applied before any comparative analysis (Methods, section 2.6). MDS, Movement Disorder Society; MDS-UPDRS, Movement Disorder Society-sponsored Unified Parkinson’s Disease Rating Scale.

Patients were eligible if they were aged 50 years or over, had a positive bedside OH screen documented during the admission (section 2.3), and had both an MDS-UPDRS Part III score and a modified H–Y stage recorded. Exclusions were atypical or secondary parkinsonism; cognitive or language impairment precluding a reliable motor examination; acute conditions known to confound orthostatic blood pressure (sepsis, acute heart failure, gastrointestinal bleeding, symptomatic anaemia); prior deep brain stimulation or device-aided therapy; and absence of the core motor variables. The exclusion of acute confounding illness is material to interpretation, since volume depletion and intercurrent illness are common alternative causes of orthostatic blood-pressure fall in hospitalized patients. Where a patient had more than one qualifying admission, only the first was analysed. Levodopa–carbidopa intestinal gel is not available in routine practice in China and no participant received it, so the device-aided exclusion operated through prior deep brain stimulation alone. Participant flow is shown in Figure 1.

### 2.3 Bedside orthostatic hypotension screening

Screening followed the departmental standard operating procedure for PD admissions and was performed by ward nursing staff using an automated oscillometric monitor. Blood pressure and heart rate were recorded after at least 5 min supine, and again at 1 min and at 3 min upright. The postural manoeuvre was active unassisted standing wherever the patient could stand safely, and assisted standing or 60° head-up tilt where they could not; because that choice is not independent of stage, the manoeuvre used is reported for every participant (Supplementary Table S1).

The screen was positive when systolic blood pressure fell by at least 20 mmHg, or diastolic by at least 10 mmHg, from the supine baseline at the 1-min or the 3-min reading. Fulfilment at either time point was sufficient. This disjunctive rule reflects the institutional procedure as actually operated, and we retain it deliberately rather than by default: transient orthostatic falls resolving within the first minute are at least as frequent in PD as classic sustained falls and carry comparable significance for orthostatic intolerance and syncope (Fanciulli et al., 2020). Both readings were available for all 125 participants, so no patient entered the cohort on an incomplete measurement, and the time point at which each met the criterion is reported (Supplementary Table S1).

A positive bedside screen is not equivalent to confirmed neurogenic OH. Tilt-table, cardiovascular reflex and catecholamine testing were not performed, and we describe the cohort throughout as screen-positive rather than as having neurogenic OH. Supine and upright blood pressure, the magnitude of the fall, the heart-rate response, documented orthostatic symptoms and repeat-confirmation status are reported in Supplementary Table S2 so that readers can judge the phenotype for themselves.

Two elements of the procedure could not be reconstructed with the precision that full reproducibility requires, and we state them rather than approximate them. Screening was performed during the morning nursing round after the morning dopaminergic dose, but timestamps were not recorded in a form that allows the post-dose interval to be determined per patient; since levodopa acutely lowers standing blood pressure and increases the proportion meeting OH criteria (Liu et al., 2023; Earl et al., 2024), variation in this interval is a source of misclassification in both directions and we treat it as a limitation (section 4.7). The make, model and calibration schedule of the monitor are likewise not recoverable from equipment records for the whole study window.

### 2.4 Variables and data sources

Data were abstracted onto a standardized case report form by two trained reviewers working independently, using definitions fixed before abstraction began. A random 10% sample was re-extracted by a third reviewer and discrepancies were adjudicated by a senior neurologist. Operational definitions and the data source for every variable are given in Supplementary Table S3. Four variables need comment here, because each carries a caveat that governs how the results may be read.

Motor severity was the MDS-UPDRS Part III total, assessed in the practically defined ON-medication state. It is therefore ON-state severity, systematically lower than OFF-state severity by a margin that itself widens with fluctuation severity. Modified H–Y stage, including sub-stages 1.5 and 2.5, was assigned by the attending neurologist.

Physician-documented sleep disturbance was coded positive when the record documented insomnia, sleep fragmentation or early-morning awakening; nocturnal akinesia, cramps or pain disrupting sleep; REM sleep behavior disorder; excessive daytime sleepiness; or nocturia explicitly recorded as interrupting sleep. Isolated nocturia without a documented sleep consequence was not counted, and no validated sleep instrument was administered. The variable is a clinician-documented binary derived from free text, not a graded measurement, and we name it accordingly rather than as “sleep quality,” which would imply a measurement of a kind we do not have.

Motor complications were a count from 0 to 8 of items on the institutional checklist (wearing-off; delayed ON or dose failure; unpredictable OFF; peak-dose dyskinesia; diphasic dyskinesia; OFF-period dystonia; freezing of gait; early-morning akinesia). This is not an MDS-UPDRS Part IV score and is not comparable with Part IV scores from other cohorts.

An anti-PD agent was a distinct pharmacological entity in Anatomical Therapeutic Chemical class N04 active on the admission medication reconciliation list. The variable counts entities, not dose intensity, and is not a proxy for the levodopa-equivalent daily dose, which could not be computed because doses were not recorded in machine-readable form across the study window. Concomitant medication with recognised orthostatic or sedative liability was abstracted by class (Supplementary Table S4). Age at motor symptom onset was patient- or informant-reported and disease duration derived from it, so both rest on recall rather than a documented diagnosis date (section 4.7).

### 2.5 Missing data

Age, sex, modified H–Y stage, MDS-UPDRS Part III, the medication reconciliation list, the motor-complication checklist, length of stay and both blood-pressure readings were complete for all 125 participants. Disease duration was available for 119 of 125 (95.2%) and age at motor symptom onset for 117 of 125 (93.6%); analyses of these use complete cases, with variable-specific denominators given in Table 1 and full completeness data in Supplementary Table S5.

**Table 1:** Baseline demographic and clinical characteristics of 125 hospitalized patients with Parkinson’s disease and a positive bedside orthostatic hypotension screen, by modified Hoehn–Yahr stage group.

Variable	Total (*n* = 125)	Early (*n* = 76)	Middle (*n* = 29)	Advanced (*n* = 20)	*p*
Age, years, mean ± SD	69.5 ± 7.4	68.6 ± 7.5	70.4 ± 5.4	71.9 ± 8.4	0.160
Age ≥ 70 years, *n* (%)	65 (52.0)	36 (47.4)	17 (58.6)	12 (60.0)	0.490
Male sex, *n* (%)	66 (52.8)	37 (48.7)	18 (62.1)	11 (55.0)	0.460
Age at motor symptom onset, years, median (IQR)^a^	64.0 (57.0–70.0)	64.5 (58.0–70.0)	63.0 (56.0–68.0)	61.5 (54.5–68.0)	0.286
Disease duration, years, median (IQR)^b^	5.0 (2.0–9.0)	3.0 (1.5–6.0)	7.0 (4.0–10.0)	10.0 (6.0–14.0)	<0.001
Hypertension, *n* (%)	65 (52.0)	40 (52.6)	13 (44.8)	12 (60.0)	0.570
Diabetes mellitus, *n* (%)	25 (20.0)	14 (18.4)	6 (20.7)	5 (25.0)	0.803
Fracture or fracture-related surgical history, *n* (%)	21 (16.8)	4 (5.3)	11 (37.9)	6 (30.0)	<0.001
Physician-documented sleep disturbance, *n* (%)	49 (39.2)	23 (30.3)	17 (58.6)	9 (45.0)	0.024
Levodopa therapy, *n* (%)	125 (100.0)	76 (100.0)	29 (100.0)	20 (100.0)	—
Number of anti-PD agents, median (IQR)	2.0 (1.0–2.0)	2.0 (1.0–2.0)	2.0 (2.0–3.0)	2.0 (2.0–3.0)	0.006
Mean ± SD^c^	1.94 ± 0.80	1.74 ± 0.59	2.31 ± 0.95	2.20 ± 0.93	—

IQR, interquartile range; PD, Parkinson’s disease; SD, standard deviation. Stage groups: early, modified Hoehn–Yahr (H–Y) 1.0–2.5; middle, H–Y 3.0; advanced, H–Y 4.0–5.0 (Methods, section 2.6). All 125 participants had a positive bedside orthostatic hypotension screen; the comparisons in this table are between H–Y stage groups within that screen-positive cohort and are not comparisons between patients with and without orthostatic hypotension. *p* values are from the Kruskal–Wallis test for continuous variables and Pearson’s *χ*^2^ test for categorical variables, with Fisher’s exact test where any expected count was below five. Variables that departed from normality on the Shapiro–Wilk test are reported as median (IQR); age was consistent with normality and is reported as mean ± SD. Operational definitions and data sources for every variable are given in Supplementary Table S3.

a *n* = 117 (93.6%); patient- or informant-reported and subject to recall error (section 4.7).

b *n* = 119 (95.2%); derived from reported year of motor symptom onset.

c Reported alongside the median because the standardized differences in Table 3 are computed from it.

For variables abstracted from free text, a record’s silence is ambiguous: an absent mention may reflect absence of the feature or absence of enquiry, and the two cannot be distinguished retrospectively. We coded absence of documentation as absence of the feature, which is conventional and which biases prevalence downward by an unquantifiable amount. This applies to sleep disturbance, fracture history and orthostatic symptoms, and it bears directly on the interpretation of the sleep findings (section 4.4).

### 2.6 Stage grouping

For the present analysis, modified H–Y stages were grouped as early (1.0–2.5), middle (3.0) and advanced (4.0–5.0). The grouping was chosen on clinical grounds and applied before any comparative analysis, but it was not registered in a protocol, and we therefore describe it as a grouping formed for this analysis rather than as prespecified. Stage 3.0 was kept separate because it marks the clinical transition from bilateral disease without postural instability to established postural instability with retained independent ambulation: the point at which fall risk becomes salient in a patient who is still walking unaided, and therefore the point at which a coexisting orthostatic fall changes in clinical meaning. Collapsing 3.0 into either neighbour would have concealed the transition of most interest. The modified H–Y scale and its sub-stages were used as described by the MDS Task Force (Goetz et al., 2004).

### 2.7 Statistical analysis

Distributional assumptions were assessed with the Shapiro–Wilk test supported by histograms and Q–Q plots. Age was consistent with normality and is summarised as mean ± SD. MDS-UPDRS Part III, the motor-complication count, the number of anti-PD agents, length of stay, disease duration and age at onset departed from normality; for these the median and interquartile range (IQR) are the primary descriptive statistics and are reported first throughout, with mean ± SD shown alongside so that the standardized differences reported later can be reconstructed by the reader.

Stage groups were compared with the Kruskal–Wallis test for continuous variables and Pearson’s *χ*^2^ test, or Fisher’s exact test where any expected count was below five, for categorical variables. Pairwise contrasts used Bonferroni-corrected Mann–Whitney *U* tests; no pairwise *p* value in this paper derives from a test on means. Effect sizes accompany every comparison: *ε*^2^ for Kruskal–Wallis tests, Cramér’s *V* for contingency tables, odds ratios for binary outcomes with the early-stage group as reference, and Spearman’s *ρ* for rank correlation. Cohen’s *d* is reported for pairwise contrasts of continuous variables as a dimensionless standardized mean difference, for comparability with a literature in which MDS-UPDRS Part III differences are conventionally expressed in these terms; it is descriptive and no inference rests on it. Interpretation thresholds were applied without exception and follow established conventions (Cohen, 1988; Sawilowsky, 2009): for Cohen’s *d*, small 0.20, medium 0.50, large 0.80 and very large ≥1.20; for *ε*^2^, small 0.01, medium 0.06 and large 0.14; and for Cramér’s *V* in a 3 × 2 table, small 0.10, medium 0.30 and large 0.50. Confidence intervals were computed analytically, and for proportions by the Wilson method.

Two outcomes were modelled to assess whether the observed stage gradients could be accounted for by age, sex, disease duration or medication exposure. Physician-documented sleep disturbance (49 events) was modelled by multivariable logistic regression with stage group, age, sex, disease duration, dopamine agonist use, any sedative-hypnotic use and antidepressant use as covariates. Fracture history (21 events) affords approximately 3.5 events per candidate covariate, at which maximum-likelihood logistic regression is unstable and liable to separation; it was therefore modelled by Firth penalized logistic regression, which produces finite, bias-reduced estimates under exactly these conditions (Firth, 1993; Heinze and Schemper, 2002), with a parsimonious covariate set. Both models are exploratory, are not corrected for multiplicity, and test the robustness of a described pattern rather than estimate causal effects. Five sensitivity analyses examined the stability of the sleep association: progressive adjustment; exclusion of benzodiazepine or Z-drug users; exclusion of diuretic or α-blocker users; and complete-case analysis for disease duration. Two-sided *p* < 0.05 was the significance threshold. Statistical analyses were performed using R version 4.4.1 (R Foundation for Statistical Computing, Vienna, Austria).

## 3 Results

### 3.1 Cohort assembly and baseline characteristics

Of 312 consecutive PD admissions during the study window, 268 underwent bedside OH screening, 161 screened positive, and 125 met all eligibility criteria and formed the analytic cohort (Figure 1). The cohort comprised 66 men (52.8%) and 59 women (47.2%), mean age 69.5 ± 7.4 years (range 50–85). Median age at motor symptom onset was 64.0 years (IQR 57.0–70.0; *n* = 117) and median disease duration 5.0 years (IQR 2.0–9.0; *n* = 119).

Disease duration rose markedly across stage groups, from a median of 3.0 years (IQR 1.5–6.0) to 7.0 (4.0–10.0) and 10.0 (6.0–14.0) (*p* < 0.001). Age at motor symptom onset did not differ (*p* = 0.286), nor did age at admission (*p* = 0.160), sex (*p* = 0.460), hypertension (*p* = 0.570) or diabetes (*p* = 0.803). Hypertension was present in 65 patients (52.0%) and did not vary by stage, underlining the coexistence of supine hypertension with orthostatic falls in this population. All 125 patients received levodopa-based therapy. Baseline characteristics are given in Table 1.

### 3.2 Stage distribution of screen-positive inpatients

**Figure 2:**
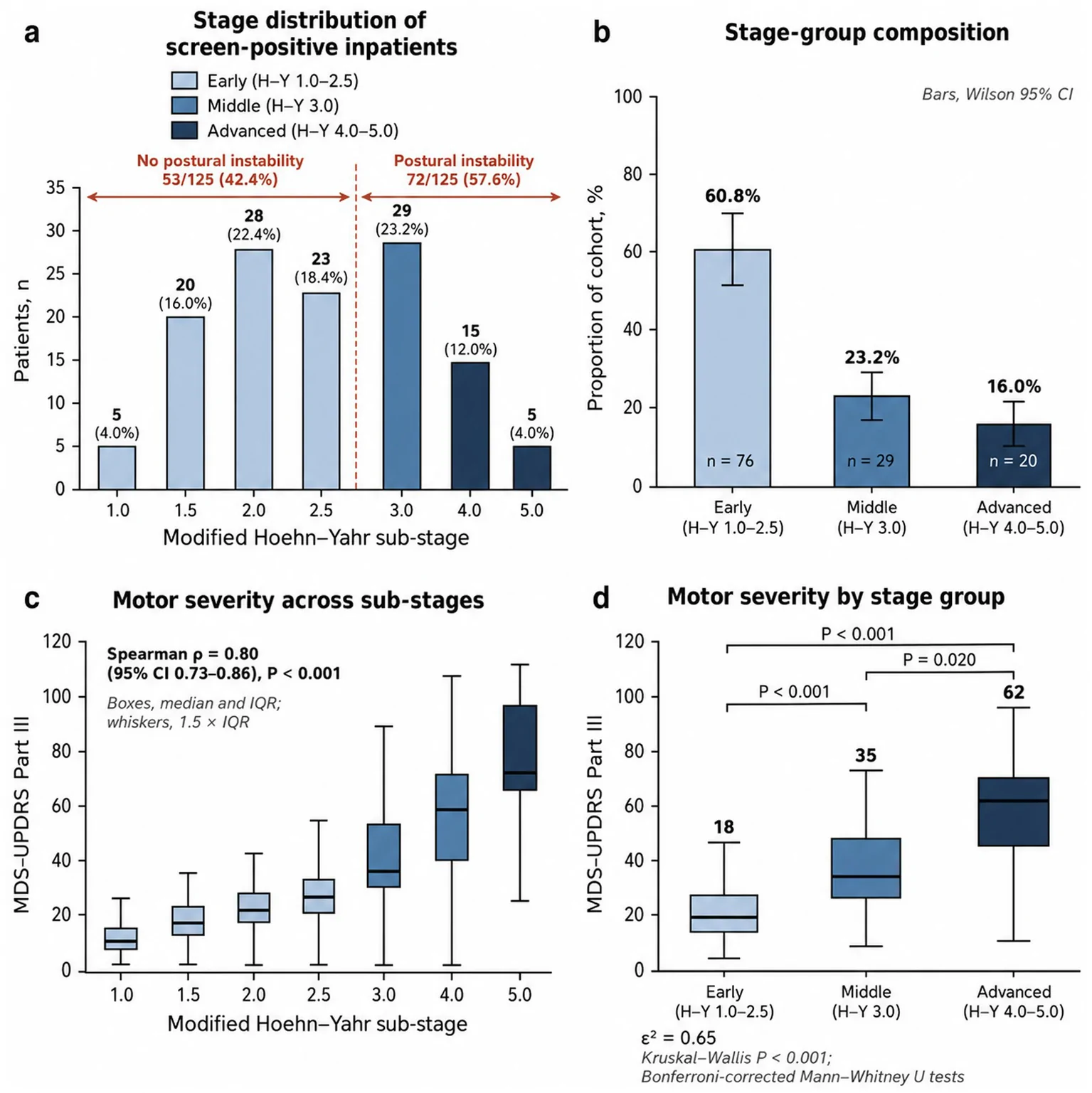
Stage distribution and motor severity of screen-positive inpatients. **(a)** Distribution across the seven modified Hoehn–Yahr (H–Y) sub-stages, colour-coded by stage group, with counts and within-cohort percentages above each bar. The dashed line marks the transition to postural instability at H–Y 3.0; 53 of 125 patients (42.4%) were screen-positive before postural instability had appeared. **(b)** Stage-group composition with Wilson 95% confidence intervals. **(c)** MDS-UPDRS Part III by sub-stage; boxes show the median and interquartile range and whiskers extend to 1.5 × IQR. Boxes are drawn from the group summary statistics reported in Supplementary Table S6. **(d)** MDS-UPDRS Part III by stage group, with Bonferroni-corrected Mann–Whitney *U p* values; no pairwise test in this panel is based on means. Group medians are annotated above each box. All comparisons are between stage groups within the screen-positive cohort.

Patients identified by the bedside screen were distributed across the entire modified H–Y spectrum rather than concentrated in advanced disease (Figure 2a). The early group accounted for 76 patients (60.8, 95% CI 52.0–68.9), the middle group for 29 (23.2, 95% CI 16.7–31.3) and the advanced group for 20 (16.0, 95% CI 10.6–23.4) (Figure 2b). Fifty-three patients (42.4, 95% CI 34.1–51.2) were at H–Y 1.0–2.0, that is, before the onset of postural instability, and the single largest sub-stage was H–Y 2.0, with 28 patients (22.4%). The full sub-stage distribution is given in Supplementary Table S6.

### 3.3 Orthostatic profile

**Figure 3:**
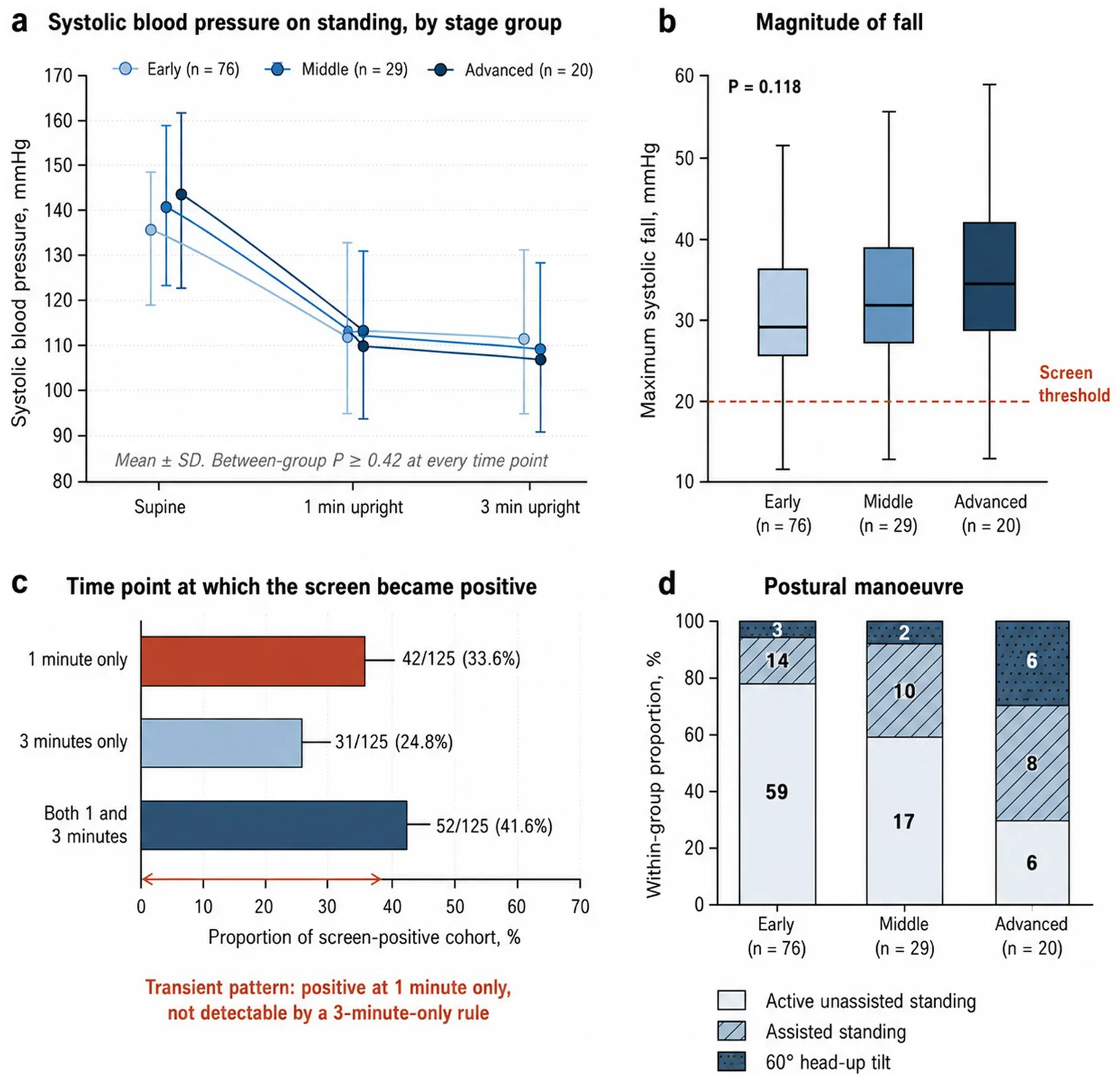
Orthostatic profile of the screen-positive cohort. **(a)** Supine, 1-min and 3-min upright systolic blood pressure by stage group (mean ± SD). Between-group differences were not significant at any time point (*p* = 0.421, *p* = 0.687, and *p* = 0.459). **(b)** Maximum systolic fall from the supine baseline; boxes show the median and interquartile range, whiskers 1.5 × IQR, and the dashed line the 20 mmHg screening threshold (Kruskal–Wallis *p* = 0.118). **(c)** Time point at which each participant met the screening criterion, with Wilson 95% confidence intervals. Forty-two patients (33.6%) met the criterion at 1 min only and would not have been identified by a screen restricted to the 3-min reading. **(d)** Postural manoeuvre used, as a within-group proportion; counts are annotated within each segment. The manoeuvre is not independent of stage and is reported for that reason. A positive bedside screen is not equivalent to confirmed neurogenic orthostatic hypotension; no tilt-table or reflex testing was performed (Methods, section 2.3). Full values are given in Supplementary Tables S1, S2.

Supine systolic blood pressure was 137.8 ± 17.6 mmHg and fell to 111.9 ± 16.5 mmHg at 1 min and 110.4 ± 16.8 mmHg at 3 min upright, with no difference across stage groups at any time point (*p* = 0.421, 0.687 and 0.459; Figure 3a). The magnitude of the fall was likewise similar across stages: the median maximum systolic fall was 27 mmHg (IQR 22–35) overall and 26, 28 and 31 mmHg across groups (*p* = 0.118; Figure 3b), and the median maximum diastolic fall 13 mmHg (IQR 10–18; *p* = 0.214). The median heart-rate increase on standing was 8 beats per minute (IQR 5–12), with a non-significant decline across stage (9, 8, 6 bpm; *p* = 0.072). Orthostatic symptoms were documented in 69 patients (55.2%), so 44.8% met the criterion without a recorded symptom, and OH was confirmed on repeat testing during the admission in 58 (46.4%). Full values are given in Supplementary Table S2.

The time point at which the screen became positive varied substantially (Figure 3c). Fifty-two patients (41.6, 95% CI 33.3–50.4) met the criterion at both 1 and 3 min; 31 (24.8, 95% CI 18.1–33.0) at 3 min only; and 42 (33.6, 95% CI 25.9–42.3) at 1 min only. This last group would not have been identified by a screen restricted to the 3-min reading. Ninety-four patients (75.2%) were positive at 1 min.

The postural manoeuvre used varied with stage, as expected (Figure 3d): active unassisted standing was used in 77.6% of the early group but 30.0% of the advanced group, while head-up tilt rose from 3.9 to 30.0%.

### 3.4 Motor severity

Median MDS-UPDRS Part III was 26.0 (IQR 15.0–41.0) for the cohort and rose steeply across stage groups: 18.0 (IQR 12.0–24.2) in the early group, 35.0 (30.0–55.0) in the middle group and 62.5 (40.0–69.2) in the advanced group (Kruskal–Wallis *p* < 0.001, *ε*^2^ = 0.65, a large effect; Table 2, Figure 2d). All three pairwise contrasts were significant, and the early-to-advanced standardized difference was very large (Cohen’s *d* = 2.79, 95% CI 2.16–3.42). Stage and motor score were strongly rank-correlated (Spearman *ρ* = 0.80, 95% CI 0.73–0.86, *p* < 0.001; Figure 2c).

**Table 2:** Motor severity, clinical burden and resource use across modified Hoehn–Yahr stage groups within the screen-positive cohort.

Indicator	Total (*n* = 125)	Early (*n* = 76)	Middle (*n* = 29)	Advanced (*n* = 20)	*p*
MDS-UPDRS Part III, median (IQR)	26.0 (15.0–41.0)	18.0 (12.0–24.2)	35.0 (30.0–55.0)	62.5 (40.0–69.2)	<0.001
Mean ± SD	30.98 ± 21.83	19.3 ± 10.5	41.8 ± 18.0	59.8 ± 24.6	—
Motor complications, *n*, median (IQR)	1.0 (0.0–4.0)	0.0 (0.0–1.0)	4.0 (2.0–7.0)	5.0 (3.0–8.0)	<0.001
Mean ± SD	2.36 ± 2.93	0.79 ± 1.59	4.40 ± 2.98	5.30 ± 2.68	—
≥1 motor complication, *n* (%)	72 (57.6)	32 (42.1)	23 (79.3)	17 (85.0)	<0.001
Length of hospital stay, days, median (IQR)	7.01 (5.10–9.07)	6.95 (5.02–8.05)	9.03 (6.99–11.07)	8.77 (5.79–14.32)	0.001
Mean ± SD	8.21 ± 3.99	7.29 ± 3.84	9.37 ± 3.27	9.99 ± 4.60	—

IQR, interquartile range; MDS-UPDRS Part III, Movement Disorder Society-sponsored Unified Parkinson’s Disease Rating Scale Part III (motor examination); SD, standard deviation. MDS-UPDRS Part III was assessed in the practically defined ON-medication state (Methods, section 2.4) and therefore reflects ON-state severity. All three indicators departed from normality on the Shapiro–Wilk test; the median (IQR) is the primary descriptive statistic and the mean ± SD is reported alongside it because the standardized differences in Table 3 are computed from it. Length of stay is markedly right-skewed and should be read from the medians. The motor-complication count is a tally of items on an institutional checklist (range 0–8) and is not an MDS-UPDRS Part IV score; it is not comparable with Part IV scores from other cohorts. *p* values are from the Kruskal–Wallis test for continuous variables and Pearson’s *χ*^2^ test for categorical variables; all pairwise contrasts referred to in the text are Bonferroni-corrected Mann–Whitney *U* tests.

Group-level separation nonetheless coexisted with substantial individual variation. The interquartile range of MDS-UPDRS Part III within the advanced group alone spanned 40.0–69.2, and scores overlapped between adjacent sub-stages throughout the range (Figure 2c). A stage label and a motor score are therefore not interchangeable at the level of the individual patient.

### 3.5 Clinical burden across stage groups

**Figure 4:**
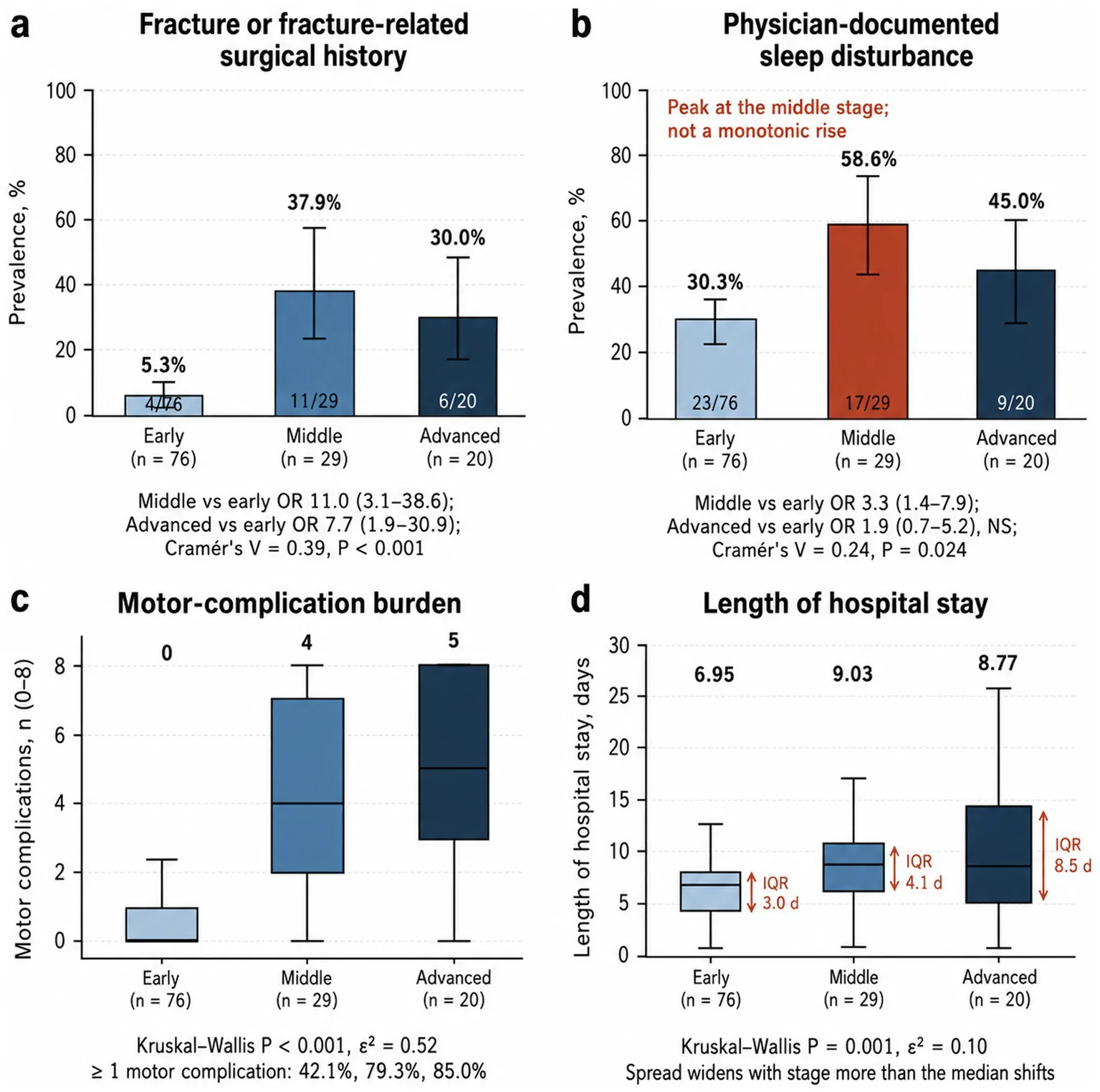
Clinical burden across modified Hoehn–Yahr stage groups. **(a)** Fracture or fracture-related surgical history, as defined in section 2.4, with Wilson 95% confidence intervals; numerators and denominators are annotated within each bar. **(b)** Physician-documented sleep disturbance, the one burden measure that does not rise monotonically with stage; the middle-stage bar is highlighted. The advanced-versus-early contrast is not significant and the middle and advanced confidence intervals overlap across almost their entire width, so these data establish a rise from early to middle disease and not a decline thereafter (section 4.4). **(c)** Motor-complication count; boxes show the median and interquartile range and whiskers 1.5 × IQR. The count is an institutional checklist tally (range 0–8) and not an MDS-UPDRS Part IV score. **(d)** Length of hospital stay; the arrows mark the interquartile range of each group. The distribution is right-skewed, the shift in median is modest, and the widening of the spread with stage is the more informative feature (section 4.5). Boxes in **(c,d)** are drawn from the group summary statistics reported in Table 2. Effect sizes for all comparisons are given in Table 3.

Fracture or fracture-related surgical history was recorded in 21 patients (16.8%) and differed sharply across stages: 4 of 76 (5.3, 95% CI 2.1–12.8), 11 of 29 (37.9, 95% CI 22.7–56.0) and 6 of 20 (30.0, 95% CI 14.5–51.9) (*p* < 0.001, Cramér’s *V* = 0.39, a medium effect; Figure 4a). Relative to the early group, the odds ratio was 11.0 (95% CI 3.1–38.6) for the middle group and 7.7 (95% CI 1.9–30.9) for the advanced group.

Motor complications increased monotonically: median counts were 0.0 (IQR 0.0–1.0), 4.0 (2.0–7.0) and 5.0 (3.0–8.0) (*p* < 0.001, *ε*^2^ = 0.52, a large effect; Figure 4c), and the proportion with at least one complication rose from 42.1 to 79.3 and 85.0% (*p* < 0.001). Pharmacotherapy burden also differed (*p* = 0.006): the median was 2.0 anti-PD agents in every group, but the mean rose to a peak at the middle rather than the advanced stage (1.74 ± 0.59, 2.31 ± 0.95, 2.20 ± 0.93).

Median length of stay was 7.01 days (IQR 5.10–9.07) overall, and 6.95 (5.02–8.05), 9.03 (6.99–11.07) and 8.77 (5.79–14.32) days across stage groups (*p* = 0.001, *ε*^2^ = 0.10, a medium effect; Figure 4d). The shift in central tendency was modest, about 2 days; the more striking feature was dispersion, with the interquartile range widening from 3.0 days in the early group to 8.5 days in the advanced group. Advanced stage was associated less with a longer stay for every patient than with the emergence of a subgroup with very long admissions.

Physician-documented sleep disturbance was the one burden measure that did not rise monotonically. It was recorded in 49 patients (39.2%) overall, and in 23 of 76 (30.3, 95% CI 21.1–41.3) early-stage, 17 of 29 (58.6, 95% CI 40.7–74.5) middle-stage and 9 of 20 (45.0, 95% CI 25.8–65.8) advanced-stage patients (*p* = 0.024, Cramér’s *V* = 0.24; Figure 4b). The middle-versus-early odds ratio was 3.3 (95% CI 1.4–7.9); the advanced-versus-early odds ratio was 1.9 (95% CI 0.7–5.2) and was not significant, with confidence intervals overlapping the middle group across almost their entire width. These data therefore establish that sleep disturbance is documented more often from the middle stage onwards than in early disease, and do not establish that it declines thereafter. Effect sizes for all comparisons are given in Table 3.

**Table 3:** Unadjusted effect sizes with 95% confidence intervals for the principal stage-dependent associations within the screen-positive cohort.

Comparison or association	Effect-size measure	Estimate (95% CI)	Interpretation	*p*
MDS-UPDRS Part III across stage groups	*ε* ^2^	0.65	Large	<0.001
MDS-UPDRS Part III, early vs. middle	Cohen’s *d*	1.73 (1.25–2.22)	Very large	<0.001
MDS-UPDRS Part III, middle vs. advanced	Cohen’s *d*	0.86 (0.27–1.46)	Large	0.020
MDS-UPDRS Part III, early vs. advanced	Cohen’s *d*	2.79 (2.16–3.42)	Very large	<0.001
H–Y stage vs. MDS-UPDRS Part III	Spearman *ρ*	0.80 (0.73–0.86)	Strong	<0.001
Motor complications across stage groups	*ε* ^2^	0.52	Large	<0.001
≥ 1 motor complication, advanced vs. early	Odds ratio	7.8 (2.1–28.9)	—	<0.001
Fracture history, middle vs. early	Odds ratio	11.0 (3.1–38.6)	—	<0.001
Fracture history, advanced vs. early	Odds ratio	7.7 (1.9–30.9)	—	0.004
Fracture history, 3 × 2 table	Cramér’s *V*	0.39	Medium	<0.001
Sleep disturbance, middle vs. early	Odds ratio	3.3 (1.4–7.9)	—	0.024
Sleep disturbance, advanced vs. early	Odds ratio	1.9 (0.7–5.2)	—	0.211
Sleep disturbance, 3 × 2 table	Cramér’s *V*	0.24	Small to medium	0.024
Anti-PD agents, early vs. middle	Cohen’s *d*	0.81 (0.37–1.25)	Large	0.006
Length of stay across stage groups	*ε* ^2^	0.10	Medium	0.001
Length of stay, early vs. advanced	Cohen’s *d*	0.67 (0.17–1.18)	Medium	0.001

CI, confidence interval; *ε*^2^, epsilon-squared (Kruskal–Wallis effect size); H–Y, modified Hoehn–Yahr; MDS-UPDRS Part III, Movement Disorder Society-sponsored Unified Parkinson’s Disease Rating Scale Part III; PD, Parkinson’s disease. Interpretation thresholds, stated here and in section 2.7 and applied without exception: Cohen’s *d*, small 0.20, medium 0.50, large 0.80, very large ≥1.20 (Cohen, 1988; Sawilowsky, 2009); *ε*^2^, small 0.01, medium 0.06, large 0.14; Cramér’s *V* for a 3 × 2 table, small 0.10, medium 0.30, large 0.50. Cohen’s *d* is a dimensionless standardized mean difference reported for comparability with the published literature; it is descriptive, no inference rests on it, and the corresponding medians are given in Table 2. Every *p* value accompanying a pairwise contrast is from a Bonferroni-corrected Mann–Whitney *U* test or, for binary outcomes, from the *χ*^2^ or Fisher’s exact test; none is from a t test. Confidence intervals were computed analytically rather than by bootstrap: for Cohen’s *d* from the pooled standard deviation and the noncentral *t* distribution, for Spearman’s *ρ* by Fisher’s *z* transformation, and for odds ratios on the log scale. Odds ratios use the early-stage group as reference, with the Haldane–Anscombe correction applied to the fracture comparisons. All estimates are unadjusted and derive from comparisons within the screen-positive cohort; no orthostatic-negative comparator was available (sections 4.1 and 4.7). Adjusted estimates are given in Table 4.

### 3.6 Medication exposure across stage groups

Because dopaminergic, sedative and antidepressant agents may influence both orthostatic blood pressure and the recording of sleep complaints, their distribution across stage groups was examined directly (Supplementary Tables S4, S7). Exposure was not uniform, but neither did it track stage in the way that a simple confounding account would predict. Dopamine agonist use peaked at the middle stage (36.8, 58.6, 45.0%) without reaching significance across groups (Cramér’s *V* = 0.18, *p* = 0.124), as did benzodiazepine or Z-drug use (11.8, 27.6, 20.0%; *V* = 0.19, *p* = 0.096) and any sedative-hypnotic use (14.5, 27.6, 25.0%; *V* = 0.17, *p* = 0.154). Antihypertensive use did not differ (*V* = 0.16, *p* = 0.206), nor did MAO-B inhibitor use (*V* = 0.09, *p* = 0.612). Only COMT inhibitor use (7.9, 27.6, 30.0%; V = 0.25, *p* = 0.021) and amantadine use (6.6, 20.7, 25.0%; *V* = 0.24, *p* = 0.029) rose significantly with stage, and neither is a recognised driver of sleep documentation.

### 3.7 Adjusted and sensitivity analyses

**Figure 5:**
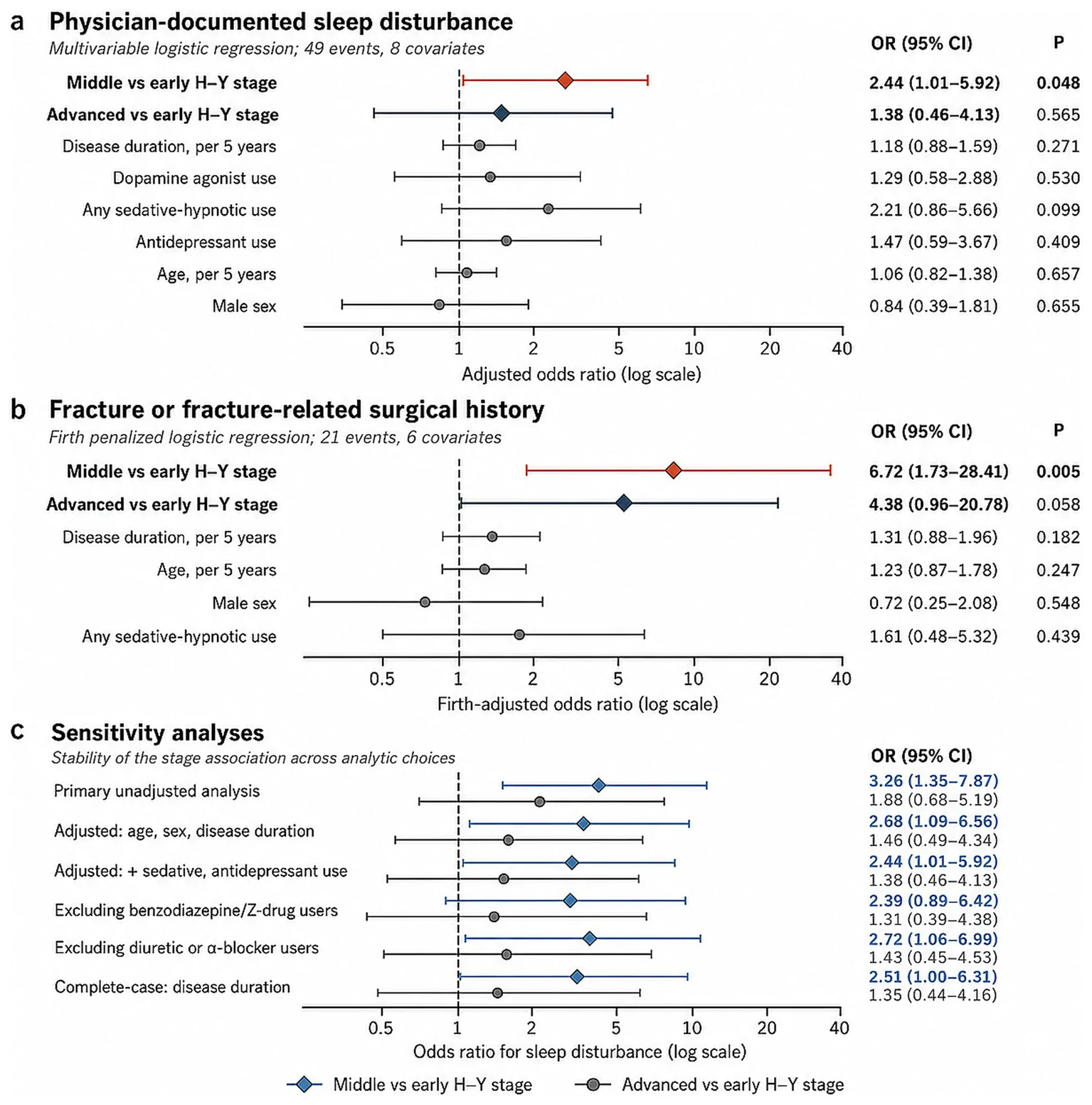
Adjusted associations and sensitivity analyses. **(a)** Multivariable logistic regression for physician-documented sleep disturbance (49 events, 8 covariates). **(b)** Firth penalized logistic regression for fracture or fracture-related surgical history (21 events, 6 covariates); penalization was used because the event count affords approximately 3.5 events per candidate covariate, at which maximum-likelihood estimation is unstable (Firth, 1993; Heinze and Schemper, 2002). In **(a,b)**, stage-group contrasts are shown as shaded rows with diamond markers and covariates as open rows with circular markers; markers are coloured to indicate a confidence interval excluding 1. **(c)** Stability of the sleep association across five sensitivity analyses; middle-versus-early and advanced-versus-early estimates are offset vertically within each row. The middle-stage estimate moves between odds ratios of 2.39 and 3.26 and loses significance only when benzodiazepine and Z-drug users are excluded, a step that removes 21 patients. All panels use a logarithmic odds-ratio axis with the null value marked by a dashed line. These models are exploratory, are not corrected for multiplicity, and are not causal estimates; because every participant was screen-positive, no model here can isolate an effect of orthostatic hypotension itself (sections 4.6 and 4.7).

In a multivariable logistic model, the middle-stage association with physician-documented sleep disturbance persisted after adjustment for age, sex, disease duration, dopamine agonist use, sedative-hypnotic use and antidepressant use (adjusted odds ratio 2.44, 95% CI 1.01–5.92, *p* = 0.048), while the advanced-stage association remained absent (1.38, 95% CI 0.46–4.13, *p* = 0.565) (Table 4, Figure 5a). No covariate was independently associated with the outcome; sedative-hypnotic use came closest (2.21, 95% CI 0.86–5.66, *p* = 0.099) and disease duration was not associated (1.18 per 5 years, 95% CI 0.88–1.59, *p* = 0.271).

**Table 4:** Adjusted associations between modified Hoehn–Yahr stage group and the two principal binary outcomes.

Predictor	Adjusted OR	95% CI	*p*
A. Physician-documented sleep disturbance: multivariable logistic regression (49 events)			
Middle vs. early H–Y stage	2.44	1.01–5.92	0.048
Advanced vs. early H–Y stage	1.38	0.46–4.13	0.565
Disease duration, per 5 years	1.18	0.88–1.59	0.271
Dopamine agonist use	1.29	0.58–2.88	0.530
Any sedative-hypnotic use	2.21	0.86–5.66	0.099
Antidepressant use	1.47	0.59–3.67	0.409
Age, per 5 years	1.06	0.82–1.38	0.657
Male sex	0.84	0.39–1.81	0.655
B. Fracture or fracture-related surgical history: Firth penalized logistic regression (21 events)			
Middle vs. early H–Y stage	6.72	1.73–28.41	0.005
Advanced vs. early H–Y stage	4.38	0.96–20.78	0.058
Disease duration, per 5 years	1.31	0.88–1.96	0.182
Age, per 5 years	1.23	0.87–1.78	0.247
Male sex	0.72	0.25–2.08	0.548
Any sedative-hypnotic use	1.61	0.48–5.32	0.439

CI, confidence interval; H–Y, modified Hoehn–Yahr; OR, odds ratio. Model A is a multivariable logistic regression including all listed covariates simultaneously; complete cases for disease duration (*n* = 119). Model B uses Firth’s penalized likelihood, which yields finite, bias-reduced estimates where events are few and separation is possible (Firth, 1993; Heinze and Schemper, 2002); the covariate set was kept deliberately parsimonious because 21 events afford approximately 3.5 events per candidate covariate. Both models are exploratory, are not corrected for multiplicity, and are reported to test whether the stage gradients described in Tables 1–3 can be accounted for by age, sex, disease duration or medication exposure. They are not causal estimates, and the absence of an orthostatic-negative comparator means that no model in this study can isolate an effect of orthostatic hypotension itself (sections 4.6 and 4.7). Sensitivity analyses for Model A are given in Figure 5c, Supplementary Table S8.

In a Firth penalized model, the middle-stage association with fracture history likewise persisted (adjusted odds ratio 6.72, 95% CI 1.73–28.41, *p* = 0.005), with the advanced-stage estimate attenuated to the margin of significance (4.38, 95% CI 0.96–20.78, *p* = 0.058) (Table 4, Figure 5b). Disease duration was not independently associated with fracture history (1.31 per 5 years, 95% CI 0.88–1.96, *p* = 0.182).

Across five sensitivity analyses the middle-stage sleep estimate was stable, moving between odds ratios of 2.39 and 3.26 and losing significance only when benzodiazepine and Z-drug users were excluded altogether (2.39, 95% CI 0.89–6.42), a step that removes 21 patients and the corresponding precision. The advanced-stage estimate remained non-significant in every analysis (Figure 5c, Supplementary Table S8).

## 4 Discussion

### 4.1 Principal findings

In 125 hospitalized PD patients identified by a routine bedside OH screen, three findings stand out. First, the screen did not select for advanced disease. Screen-positive patients spanned the whole modified H–Y spectrum, and 42.4% were at stage 1.0–2.0, before postural instability had appeared. Second, a third of the cohort met the OH criterion at 1 min only, and would have been missed by a screen restricted to the classic 3-min reading. Third, within this screen-positive group, motor severity, motor complications, fracture history and length of stay all increased with stage, while physician-documented sleep disturbance instead peaked at the middle stage and remained elevated there after adjustment.

Because every participant was screen-positive by construction, these comparisons describe the population a bedside screen identifies. They cannot show, and we do not claim, that the screening result marks burden independently of stage.

### 4.2 A bedside screen identifies patients across the disease spectrum

The stage distribution is the finding with the most direct bearing on practice. Had screen-positive patients clustered in advanced disease, a positive screen would have added little to a stage assessment the clinician has already made. That is not what we observed. The largest single sub-stage was H–Y 2.0, and over 40% of the cohort had no postural instability at all. This is consistent with the meta-analytic finding that although patients with PD and OH are older and have longer disease duration than those without, the difference in duration is small, with a weighted mean difference of only 0.71 years (Wang et al., 2025), and with prospective data showing appreciable OH prevalence in early and *de novo* PD (Mei et al., 2024). A bedside screen, in other words, identifies a group that stage alone does not predict.

The corollary matters for what follows: because the screen selects across the spectrum, the burden a clinician should anticipate on a positive screen depends heavily on where the patient sits along it, and the gradients described below are the practical content of that dependence.

### 4.3 Transient orthostatic falls and the timing of measurement

That 33.6% of our cohort met the criterion at 1 min only is, to our knowledge, the first description of this pattern in a consecutively screened PD inpatient population, and it has an immediate practical implication: a ward protocol that measures blood pressure only at 3 min would have classified a third of these patients as normal. The finding aligns with continuous-monitoring data showing that transient OH, resolving within the first minute of standing, is at least as common in PD as classic sustained OH and is associated with orthostatic intolerance and syncope, while being readily overlooked by intermittent bedside measurement (Fanciulli et al., 2020). Our data extend that observation from an autonomic laboratory to routine ward practice with nothing more than an oscillometric cuff and a second reading.

Two cautions apply. Intermittent oscillometric measurement at 1 min cannot distinguish initial orthostatic hypotension, a physiological transient of the first 15 to 30 s, from an early sustained fall; continuous beat-to-beat monitoring would be required (Fanciulli et al., 2020). And a 1-min-only fall may in some patients be an artefact rather than a reproducible finding, a caveat underlined by the 46.4% repeat-confirmation rate, though repeat testing was not systematic and its absence cannot be read as non-confirmation. What our data support is a narrow and useful claim: restricting a bedside screen to 3 min discards information that a 1-min reading captures at no additional cost.

Interpretation of the orthostatic profile should otherwise remain modest. Neither supine nor upright pressure nor the magnitude of the fall differed across stage groups, indicating that stage-related burden here is not explained by more severe orthostatic falls in more advanced patients. The blunted heart-rate response (median 8 bpm) is compatible with a neurogenic mechanism in a substantial fraction, but we did not perform the testing that would establish it.

### 4.4 Why sleep disturbance peaks at the middle stage

Sleep disturbance was the only burden measure that did not rise with stage, and the pattern survived adjustment. Three explanations deserve consideration, and these data cannot adjudicate between them.

The first is statistical and should be dealt with before any biology is invoked. The advanced group contains 20 patients, its confidence interval (25.8–65.8%) overlaps the middle group’s (40.7–74.5%) across almost its entire width, and the advanced-versus-early contrast is null both unadjusted and adjusted. These data establish a rise from early to middle disease, not a fall thereafter.

The second is ascertainment, and it is the explanation we find most persuasive. Sleep disturbance was recorded only when a clinician documented it, which requires that a patient raised the complaint. Non-reporting of non-motor symptoms in PD is not marginal: up to 72% of patients with a distressing non-motor symptom have never raised it with a healthcare provider, the commonest barrier being acceptance of the symptom as an inevitable part of the disease (Hurt et al., 2019), a barrier that plausibly hardens with disease duration. A prevalence curve that rises then falls is precisely what a detection process degrading with severity would produce on top of a true prevalence that rises monotonically. Instrumented data support this reading: in 448 Chinese patients with advanced PD assessed with the PDSS-2 rather than by chart review, significant nocturnal disturbance was present in 70.9% (95% CI 66.7–75.1) and rose with H–Y stage, with an odds ratio of 1.36 (95% CI 1.06–1.74) per stage and no hint of a plateau (He et al., 2021). When the measurement improves, the peak disappears.

The third is biological and, unlike the others, would predict a real maximum. Insomnia in PD is driven substantially by nocturnal motor phenomena, namely nocturnal akinesia, inability to turn in bed, OFF-period dystonia and early-morning akinesia (Falup-Pecurariu et al., 2026), whose impact peaks once the therapeutic window has narrowed but before mobility is lost. Our data fit that timing, since both the anti-PD agent count and the steep rise in motor complications occur in the middle group. A patient at H–Y 3.0 is still ambulatory, still fluctuating and still attempting to turn in bed unaided, so every nocturnal OFF phenomenon is a disruptive event; a patient at H–Y 4.0 or 5.0 may be immobile enough, and assisted closely enough at night, that the same pathophysiology no longer generates a reportable complaint. Longitudinal evidence also cautions against expecting a smooth gradient, since insomnia in PD appears and remits within individuals rather than accumulating steadily (Zhu et al., 2016).

Distinguishing these requires a better instrument, not a new cohort: the PDSS-2 (Trenkwalder et al., 2011) or the Pittsburgh Sleep Quality Index (Buysse et al., 1989) administered prospectively to patient and caregiver separately would separate a true maximum from a documentation artefact within a single admission. The clinical implication is the same under all three: nocturnal symptoms merit active enquiry at every stage and should not be assumed absent because they are not in the notes.

### 4.5 Fractures, motor complications and resource use

The fracture gradient was the steepest association we observed and survived penalized adjustment, with a more than sixfold increase in odds from early to middle stage. Its shape is instructive: the peak is at the middle rather than the advanced stage, which is what would be expected if exposure to falling requires both postural instability and retained ambulation. A patient at H–Y 3.0 has both; a patient at H–Y 5.0 has only the first. Postural instability combined with an orthostatic fall in a patient still walking unaided is a plausible mechanical explanation, and it identifies stage 3.0 as the point of maximum preventable risk, consistent with evidence that OH is a modifiable risk factor for falls in PD (Zhang et al., 2024; Fanciulli et al., 2020; LeWitt et al., 2020).

The accrual of motor complications with stage is expected from the natural history of long-term levodopa therapy (Aradi and Hauser, 2020). Its relevance here is that it locates the middle stage as the point at which fluctuation and regimen complexity first rise steeply, coinciding with both the fracture and the sleep peaks.

The length-of-stay finding should be read from the medians and read modestly: the increment is about two days. The widening of the distribution is more informative, since it is the tail rather than the average that drives bed occupancy.

Hypertension was present in over half the cohort and did not vary by stage, a reminder that supine hypertension and orthostatic falls coexist and that treating one can worsen the other (Fanciulli et al., 2025; Grosu et al., 2025). Any pharmacological response to a positive screen must be weighed against that background rather than against the orthostatic reading alone.

### 4.6 Medication as an explanation

Dopaminergic, sedative and antidepressant agents are frequently invoked to explain associations of this kind, and their distribution is reported in full (Supplementary Tables S4, S7) so that readers may judge it directly. Three considerations bear on how much weight the explanation can carry.

Structurally, OH here was an eligibility criterion rather than an outcome. Every comparison is between stage groups within a cohort in which OH status is constant, so medication cannot distort an association between OH and burden that we never estimate; what it could plausibly do is influence entry into the cohort, which is a question about selection.

Empirically, the exposure data do not fit a straightforward confounding account. The agents most often implicated in sleep documentation, dopamine agonists and sedative-hypnotics, peaked at the middle stage rather than rising with stage, and neither reached significance across groups. The two classes that did rise significantly, COMT inhibitors and amantadine, are not recognised drivers of sleep complaints. Adjustment attenuated the middle-stage estimate from 3.26 to 2.44 without abolishing it.

Finally, the evidence that dopaminergic agents cause OH is weaker than is often assumed. In a meta-analysis of randomized trials, neither dopamine agonists (odds ratio 1.39, 95% CI 0.97–1.98) nor MAO-B inhibitors (2.28, 95% CI 0.81–6.46) were significantly associated with OH relative to placebo, and OH was so inconsistently defined that the drug-attributable fraction could not be estimated (Nimmons et al., 2022); the evidence for non-parkinsonian agents is stronger, with the highest odds for beta-blockers and tricyclic antidepressants (Bhanu et al., 2021), which is why those classes are reported separately. Our position is neither that medication explains these findings nor that it can be dismissed: the effect is plausible, its magnitude here is unquantified, and a cohort in which every patient is OH-positive by construction cannot quantify it.

### 4.7 Strengths and limitations

The principal strengths are the consecutive design with a documented flow from 312 admissions to 125 participants, the exclusion of acute illness known to confound orthostatic blood pressure, a validated motor scale and neurologist-assigned staging, duplicate abstraction against definitions fixed in advance, complete 1- and 3-min readings for every participant, and effect sizes with confidence intervals reported throughout rather than *p* values alone.

The limitations are substantial and shape what may be concluded. First and most important, there is no PD comparison group without OH: every finding is a within-screen-positive comparison, and none can establish that a positive screen marks burden independently of stage. This is structural rather than remediable by analysis. Second, a positive bedside screen is not confirmed neurogenic OH; no tilt-table or reflex testing was performed and, although acute confounders were an explicit exclusion and supine, upright and heart-rate data are reported, some participants will have had non-neurogenic falls. Third, the post-dose interval at which screening occurred could not be reconstructed, and since levodopa acutely lowers standing blood pressure and increases the proportion meeting OH criteria (Liu et al., 2023; Earl et al., 2024), patients screened nearer the peak effect were more likely to enter the cohort.

Fourth, the levodopa-equivalent daily dose could not be computed, so pharmacotherapy burden is represented only by a count of agents, and disease duration and age at onset rest on recall. Fifth, ascertainment quality is not uniform: motor scores, staging, medications and length of stay came from structured fields, whereas sleep disturbance, fracture history and age at onset came from free text. Sleep disturbance in particular is a clinician-documented binary aggregating several distinct phenomena, and its middle-stage peak may be a property of that ascertainment rather than of the disease (section 4.4); fractures could not be subclassified as fall-related, which is precisely the subgroup of most interest here. Sixth, the adjusted models are exploratory, with roughly six events per covariate for sleep and only 21 events for fracture, no correction for multiplicity, and wide confidence intervals. Finally, this is a single centre in one Chinese province, motor severity was assessed only in the ON state, and no patient was followed beyond discharge.

### 4.8 Implications and future directions

Two implications follow that do not depend on resolving the questions this design leaves open. A bedside orthostatic screen in PD inpatients should include a 1-min reading, because a third of the patients it detects are detectable at no other time point and at no additional cost. And a positive screen should not be read as a marker of advanced disease, because two in five of the patients it identifies have no postural instability at all.

The questions that remain require a different study, and its design follows from the limitations above: consecutive PD inpatients enrolled irrespective of screening result, so that a screen-negative comparator exists; orthostatic assessment at a fixed, recorded post-dose interval with tilt-table confirmation in a subset; disease duration recorded prospectively; the levodopa-equivalent daily dose computed by a published conversion (Tomlinson et al., 2010) with every OH-relevant co-prescription as a prespecified covariate; sleep assessed by instrument rather than chart review; and follow-up for falls, fractures, readmission and mortality. Building on prospective work such as the 7-year population-based study of Hiorth et al. (2019), such a design would establish whether a bedside screen carries prognostic value of its own. We regard the present study as the descriptive groundwork for that work rather than a substitute for it.

## 5 Conclusion

Hospitalized patients with Parkinson’s disease and a positive bedside orthostatic hypotension screen are not a uniformly advanced group. They span the entire modified Hoehn–Yahr spectrum, two in five have no postural instability, and a third are identifiable only by a 1-min reading that a conventional 3-min protocol would omit. Within this screen-positive group, motor severity, motor complications, fracture history and length of stay increase with stage, with effect sizes from medium to very large, while physician-documented sleep disturbance peaks at the middle stage and persists there after adjustment for age, sex, disease duration and medication exposure.

Because every participant was screen-positive and no orthostatic-negative comparator was available, all comparisons reported here are between stage groups within the screen-positive cohort. They describe the population a routine ward screen identifies; they do not establish that the screening result marks clinical burden independently of disease stage. Testing that question requires a comparator, a fixed post-dose testing interval, adjustment for levodopa-equivalent dose and longitudinal follow-up.

## Data Availability

The original contributions presented in the study are included in the article/Supplementary material, further inquiries can be directed to the corresponding author.
