# Supplementary material for: Stage-dependent clinical burden in hospitalized Parkinson’s disease with a positive bedside orthostatic hypotension screen: a retrospective cross-sectional analysis
Source: Front Aging Neurosci. 2026 Aug 12;18:1894279. doi: 10.3389/fnagi.2026.1894279 (PMC13506841; doi:10.3389/fnagi.2026.1894279)
Supplement: Supplementary file 1 [file Data_sheet_1.docx]

**Supplementary Material**

**Supplementary Table S1.** Postural manoeuvre and time point of screen positivity, by modified Hoehn–Yahr stage group. Values are n (%) of the group.

| **Variable** | **Total (n = 125)** | **Early (n = 76)** | **Middle (n = 29)** | **Advanced (n = 20)** |
| --- | --- | --- | --- | --- |
| **POSTURAL MANOEUVRE** | | | | |
| Active unassisted standing | 82 (65.6) | 59 (77.6) | 17 (58.6) | 6 (30.0) |
| Assisted standing | 32 (25.6) | 14 (18.4) | 10 (34.5) | 8 (40.0) |
| 60° head-up tilt | 11 (8.8) | 3 (3.9) | 2 (6.9) | 6 (30.0) |
| **TIME POINT OF SCREEN POSITIVITY** | | | | |
| 1 minute only | 42 (33.6) | 28 (36.8) | 9 (31.0) | 5 (25.0) |
| 3 minutes only | 31 (24.8) | 20 (26.3) | 7 (24.1) | 4 (20.0) |
| Both 1 and 3 minutes | 52 (41.6) | 28 (36.8) | 13 (44.8) | 11 (55.0) |
| Positive at 1 minute (any) | 94 (75.2) | 56 (73.7) | 22 (75.9) | 16 (80.0) |
| Positive at 3 minutes (any) | 83 (66.4) | 48 (63.2) | 20 (69.0) | 15 (75.0) |

*Both the 1-minute and the 3-minute readings were available for all 125 participants. The postural manoeuvre was active unassisted standing wherever the patient could stand safely, and assisted standing or head-up tilt where they could not; the choice is therefore not independent of stage and is reported for that reason. The 42 patients positive at 1 minute only (33.6% of the cohort) would not have been identified by a screen restricted to the 3-minute reading. Column percentages within each block sum to 100 for the manoeuvre and time-point blocks; the two summary rows overlap by design and do not.*

**Supplementary Table S2.** Orthostatic vital-sign profile, by modified Hoehn–Yahr stage group.

| **Variable** | **Total (n = 125)** | **Early (n = 76)** | **Middle (n = 29)** | **Advanced (n = 20)** | **P** |
| --- | --- | --- | --- | --- | --- |
| Supine SBP, mmHg, mean ± SD | 137.8 ± 17.6 | 136.4 ± 16.9 | 139.1 ± 17.2 | 141.2 ± 20.4 | 0.421 |
| 1-min upright SBP, mmHg, mean ± SD | 111.9 ± 16.5 | 112.8 ± 15.9 | 111.3 ± 16.3 | 109.4 ± 19.1 | 0.687 |
| 3-min upright SBP, mmHg, mean ± SD | 110.4 ± 16.8 | 111.6 ± 16.2 | 109.7 ± 16.4 | 106.9 ± 19.2 | 0.459 |
| Maximum SBP fall, mmHg, median (IQR) | 27 (22–35) | 26 (22–33) | 28 (23–36) | 31 (24–40) | 0.118 |
| Maximum DBP fall, mmHg, median (IQR) | 13 (10–18) | 12 (10–17) | 14 (10–19) | 15 (11–20) | 0.214 |
| Heart-rate increase, bpm, median (IQR) | 8 (5–12) | 9 (6–13) | 8 (5–11) | 6 (3–10) | 0.072 |
| Orthostatic symptoms documented, n (%) | 69 (55.2) | 39 (51.3) | 17 (58.6) | 13 (65.0) | 0.486 |
| Repeat OH confirmation during admission, n (%) | 58 (46.4) | 31 (40.8) | 16 (55.2) | 11 (55.0) | 0.315 |

*DBP, diastolic blood pressure; IQR, interquartile range; OH, orthostatic hypotension; SBP, systolic blood pressure; SD, standard deviation. Neither the absolute pressures nor the magnitude of the orthostatic fall differed across stage groups, indicating that stage-related burden in this cohort is not explained by more severe orthostatic falls in more advanced patients. Orthostatic symptoms were absent from the record in 44.8% of participants, and repeat testing was performed at clinical discretion rather than systematically, so a non-confirmed screen cannot be read as a refuted one. These data describe a positive bedside screen and do not establish neurogenic orthostatic hypotension, for which tilt-table or reflex testing would be required.*

**Supplementary Table S3.** Operational definitions and data sources for every study variable.

| **Variable** | **Operational definition** | **Data source** |
| --- | --- | --- |
| Modified Hoehn–Yahr stage | Stage 1.0–5.0 including sub-stages 1.5 and 2.5, assigned by the attending neurologist during the index admission | Structured field |
| MDS-UPDRS Part III | Total motor examination score, practically defined ON-medication state | Structured field |
| Positive bedside OH screen | Fall ≥ 20 mmHg systolic or ≥ 10 mmHg diastolic from supine baseline at the 1-minute or the 3-minute reading | Nursing observation chart |
| Physician-documented sleep disturbance | Any documented: insomnia; sleep fragmentation or early-morning awakening; nocturnal akinesia, cramps or pain disrupting sleep; REM sleep behaviour disorder; excessive daytime sleepiness; nocturia explicitly recorded as interrupting sleep. Isolated nocturia without documented sleep consequence not counted | Free text |
| Fracture or fracture-related surgical history | Any documented fracture at any skeletal site after PD diagnosis, or an operation to treat one. Pathological fractures due to malignancy excluded. Mechanism not consistently documented, so not subclassified as fall-related | Free text and coded diagnoses |
| Motor complications | Count 0–8 of institutional checklist items: wearing-off; delayed ON or dose failure; unpredictable OFF; peak-dose dyskinesia; diphasic dyskinesia; OFF-period dystonia; freezing of gait; early-morning akinesia. Not an MDS-UPDRS Part IV score | Structured checklist |
| Number of anti-PD agents | Count of distinct ATC class N04 entities active on the admission reconciliation list. Fixed-dose combinations and different formulations of one molecule counted once. Counts entities, not dose intensity | Medication reconciliation |
| Concomitant medication classes | Antihypertensive (beta-blocker, calcium-channel blocker, ACE inhibitor or ARB, diuretic, α-blocker), benzodiazepine or Z-drug, sedating antihistamine, antidepressant, antipsychotic, active on the admission reconciliation list | Medication reconciliation |
| Age at motor symptom onset | Patient- or informant-reported year of first motor symptom | Free text |
| Disease duration | Interval from reported year of motor symptom onset to index admission | Derived |
| Comorbidities | Hypertension, diabetes mellitus, from coded discharge diagnoses | Coded diagnoses |
| Length of hospital stay | Discharge date minus admission date, in days | Administrative field |

*ACE, angiotensin-converting enzyme; ARB, angiotensin-receptor blocker; ATC, Anatomical Therapeutic Chemical; MDS-UPDRS, Movement Disorder Society-sponsored Unified Parkinson’s Disease Rating Scale; OH, orthostatic hypotension; PD, Parkinson’s disease; REM, rapid eye movement. Definitions were fixed before abstraction began. Variables drawn from free text are less reliable than those from structured fields, and for these an absence of documentation was coded as absence of the feature, which biases prevalence downward by an unquantifiable amount.*

**Supplementary Table S4.** Medication exposure at admission, by modified Hoehn–Yahr stage group. Values are n (%) of the group.

| **Variable** | **Total (n = 125)** | **Early (n = 76)** | **Middle (n = 29)** | **Advanced (n = 20)** |
| --- | --- | --- | --- | --- |
| **ANTI-PARKINSONIAN THERAPY** | | | | |
| Levodopa preparation | 125 (100.0) | 76 (100.0) | 29 (100.0) | 20 (100.0) |
| Dopamine agonist | 54 (43.2) | 28 (36.8) | 17 (58.6) | 9 (45.0) |
| MAO-B inhibitor | 31 (24.8) | 18 (23.7) | 9 (31.0) | 4 (20.0) |
| COMT inhibitor | 20 (16.0) | 6 (7.9) | 8 (27.6) | 6 (30.0) |
| Amantadine | 16 (12.8) | 5 (6.6) | 6 (20.7) | 5 (25.0) |
| Anticholinergic | 3 (2.4) | 2 (2.6) | 1 (3.4) | 0 (0.0) |
| **CONCOMITANT MEDICATION WITH ORTHOSTATIC LIABILITY** | | | | |
| Any antihypertensive | 54 (43.2) | 31 (40.8) | 11 (37.9) | 12 (60.0) |
| Beta-blocker | 13 (10.4) | 8 (10.5) | 2 (6.9) | 3 (15.0) |
| Calcium-channel blocker | 25 (20.0) | 15 (19.7) | 5 (17.2) | 5 (25.0) |
| ACE inhibitor or ARB | 30 (24.0) | 18 (23.7) | 6 (20.7) | 6 (30.0) |
| Diuretic | 13 (10.4) | 5 (6.6) | 3 (10.3) | 5 (25.0) |
| α-blocker | 7 (5.6) | 3 (3.9) | 2 (6.9) | 2 (10.0) |
| **CONCOMITANT MEDICATION WITH SEDATIVE LIABILITY** | | | | |
| Benzodiazepine or Z-drug | 21 (16.8) | 9 (11.8) | 8 (27.6) | 4 (20.0) |
| Sedating antihistamine | 5 (4.0) | 3 (3.9) | 1 (3.4) | 1 (5.0) |
| Any sedative-hypnotic | 24 (19.2) | 11 (14.5) | 8 (27.6) | 5 (25.0) |
| Antidepressant, any | 24 (19.2) | 11 (14.5) | 8 (27.6) | 5 (25.0) |
| of which tricyclic | 4 (3.2) | 1 (1.3) | 2 (6.9) | 1 (5.0) |
| of which SSRI or SNRI | 20 (16.0) | 10 (13.2) | 6 (20.7) | 4 (20.0) |
| Antipsychotic | 10 (8.0) | 3 (3.9) | 4 (13.8) | 3 (15.0) |

*ACE, angiotensin-converting enzyme; ARB, angiotensin-receptor blocker; COMT, catechol-O-methyltransferase; MAO-B, monoamine oxidase B; SNRI, serotonin–noradrenaline reuptake inhibitor; SSRI, selective serotonin reuptake inhibitor. Exposure reflects agents active on the admission medication reconciliation list. Beta-blockers and tricyclic antidepressants are reported separately because they carry the highest reported odds of drug-induced orthostatic hypotension. Formal comparisons across stage groups are given in Supplementary Table S7.*

**Supplementary Table S5.** Data completeness by variable.

| **Variable** | **Available, n/N** | **Completeness, %** |
| --- | --- | --- |
| Age and sex | 125/125 | 100.0 |
| Modified Hoehn–Yahr stage | 125/125 | 100.0 |
| MDS-UPDRS Part III | 125/125 | 100.0 |
| Complete 1- and 3-minute blood-pressure readings | 125/125 | 100.0 |
| Medication reconciliation | 125/125 | 100.0 |
| Motor-complication checklist | 125/125 | 100.0 |
| Sleep documentation | 125/125 | 100.0 |
| Fracture history documentation | 125/125 | 100.0 |
| Length of hospital stay | 125/125 | 100.0 |
| Disease duration | 119/125 | 95.2 |
| Age at motor symptom onset | 117/125 | 93.6 |

*MDS-UPDRS, Movement Disorder Society-sponsored Unified Parkinson’s Disease Rating Scale. Analyses of disease duration and age at motor symptom onset use complete cases; a complete-case sensitivity analysis for disease duration is given in Supplementary Table S8. For variables abstracted from free text, completeness denotes that the record was reviewed, not that the feature was enquired after: an absent mention was coded as absence of the feature and may instead reflect absence of enquiry.*

**Supplementary Table S6.** Distribution of modified Hoehn–Yahr sub-stages and motor severity within each sub-stage.

| **Modified H–Y sub-stage** | **n (%)** | **Stage group** | **MDS-UPDRS Part III, median (IQR)** |
| --- | --- | --- | --- |
| 1.0 | 5 (4.0) | Early | 9 (5–13) |
| 1.5 | 20 (16.0) | Early | 15 (10–20) |
| 2.0 | 28 (22.4) | Early | 20 (15–26) |
| 2.5 | 23 (18.4) | Early | 24 (18–30) |
| 3.0 | 29 (23.2) | Middle | 35 (30–55) |
| 4.0 | 15 (12.0) | Advanced | 58 (40–68) |
| 5.0 | 5 (4.0) | Advanced | 70 (68–102) |
| Total | 125 (100.0) | — | 26.0 (15.0–41.0) |

*H–Y, Hoehn–Yahr; IQR, interquartile range; MDS-UPDRS, Movement Disorder Society-sponsored Unified Parkinson’s Disease Rating Scale. Fifty-three patients (42.4%) were at sub-stage 1.0 to 2.0, that is, before the appearance of postural instability. The single largest sub-stage was 2.0. Motor scores overlap between adjacent sub-stages throughout the range, so a stage label and a motor score are not interchangeable at the level of the individual patient.*

**Supplementary Table S7.** Medication-exposure comparisons across modified Hoehn–Yahr stage groups.

| **Exposure** | **Cramér’s V** | **P** | **Pattern across stage groups** |
| --- | --- | --- | --- |
| Dopamine agonist use | 0.18 | 0.124 | Peaks at middle stage |
| MAO-B inhibitor use | 0.09 | 0.612 | No trend |
| COMT inhibitor use | 0.25 | 0.021 | Rises with stage |
| Amantadine use | 0.24 | 0.029 | Rises with stage |
| Any antihypertensive | 0.16 | 0.206 | No trend |
| Benzodiazepine or Z-drug use | 0.19 | 0.096 | Peaks at middle stage |
| Any sedative-hypnotic use | 0.17 | 0.154 | Peaks at middle stage |

*COMT, catechol-O-methyltransferase; MAO-B, monoamine oxidase B. Cramér’s V for a 3 × 2 contingency table; thresholds small 0.10, medium 0.30, large 0.50. The agents most often invoked to explain sleep documentation, namely dopamine agonists and sedative-hypnotics, peak at the middle stage rather than rising with stage, and neither reaches significance across groups. The two classes that do rise significantly, COMT inhibitors and amantadine, are not recognised drivers of sleep complaints. These comparisons are exploratory and are not corrected for multiplicity.*

**Supplementary Table S8.** Sensitivity analyses for the association between modified Hoehn–Yahr stage group and physician-documented sleep disturbance.

| **Analysis** | **Middle vs early, OR (95% CI)** | **Advanced vs early, OR (95% CI)** |
| --- | --- | --- |
| Primary unadjusted analysis | 3.26 (1.35–7.87) | 1.88 (0.68–5.19) |
| Adjusted for age, sex and disease duration | 2.68 (1.09–6.56) | 1.46 (0.49–4.34) |
| Further adjusted for sedative-hypnotic and antidepressant use | 2.44 (1.01–5.92) | 1.38 (0.46–4.13) |
| Excluding benzodiazepine or Z-drug users (n = 21 removed) | 2.39 (0.89–6.42) | 1.31 (0.39–4.38) |
| Excluding diuretic or α-blocker users | 2.72 (1.06–6.99) | 1.43 (0.45–4.53) |
| Complete-case analysis for disease duration (n = 119) | 2.51 (1.00–6.31) | 1.35 (0.44–4.16) |

*CI, confidence interval; OR, odds ratio. The middle-stage estimate moves between 2.39 and 3.26 across all six analyses and loses conventional significance only when benzodiazepine and Z-drug users are excluded altogether, a step that removes 21 patients and the corresponding precision rather than reversing the direction of effect. The advanced-versus-early estimate is non-significant in every analysis. These analyses are exploratory, are not corrected for multiplicity, and are not causal estimates; because every participant was screen-positive, none can isolate an effect of orthostatic hypotension itself.*

**Supplementary File 1. STROBE checklist**

The STROBE checklist for cross-sectional studies, completed with the page and line numbers of the submitted manuscript, is provided as a separate file (STROBE_checklist.docx) in the format required by the journal.
