# Supplementary material for: Stage-dependent clinical burden in hospitalized Parkinson’s disease with a positive bedside orthostatic hypotension screen: a retrospective cross-sectional analysis
Source: Front Aging Neurosci. 2026 Aug 12;18:1894279. doi: 10.3389/fnagi.2026.1894279 (PMC13506841; doi:10.3389/fnagi.2026.1894279)
Supplement: Supplementary file 2 [file Table_1.docx]

**Supplementary File: STROBE Statement Checklist**

This checklist documents how the present manuscript addresses each item of the STROBE statement for cohort studies. Page numbers refer to the main manuscript file.

| **Section** | **Item** | **Recommendation** | **Page / Section** |
| --- | --- | --- | --- |
| Title and abstract | 1(a) | Indicate the study's design with a commonly used term in the title or the abstract | Title |
|  | 1(b) | Provide in the abstract an informative and balanced summary of what was done and what was found | Abstract (p.1) |
| **Introduction** |  |  |  |
| Background/rationale | 2 | Explain the scientific background and rationale for the investigation being reported | Introduction (pp.2-3) |
| Objectives | 3 | State specific objectives, including any prespecified hypotheses | Introduction (p.3) |
| **Methods** |  |  |  |
| Study design | 4 | Present key elements of study design early in the paper | §2.1 (p.3) |
| Setting | 5 | Describe the setting, locations, and relevant dates, including periods of recruitment, exposure, follow-up, and data collection | §2.1 (p.3) |
| Participants | 6(a) | Cohort study—Give the eligibility criteria, and the sources and methods of selection of participants | §2.2 (pp.3-4) |
| Variables | 7 | Clearly define all outcomes, exposures, predictors, potential confounders, and effect modifiers. Give diagnostic criteria, if applicable | §2.3-2.5 (p.4) |
| Data sources/measurement | 8 | For each variable of interest, give sources of data and details of methods of assessment (measurement) | §2.3, §2.4, §2.5 (p.4) |
| Bias | 9 | Describe any efforts to address potential sources of bias | §2.3 (double extraction); §4.3 |
| Study size | 10 | Explain how the study size was arrived at | §2.2 (consecutive eligible inpatients) |
| Quantitative variables | 11 | Explain how quantitative variables were handled in the analyses. If applicable, describe which groupings were chosen and why | §2.5, §2.6 (pp.4-5) |
| Statistical methods | 12(a) | Describe all statistical methods, including those used to control for confounding | §2.6 (p.5) |
|  | 12(b) | Describe any methods used to examine subgroups and interactions | §2.6 (stage-stratified analyses) |
|  | 12(c) | Explain how missing data were addressed | §2.2 (missing core data excluded) |
|  | 12(d) | If applicable, explain how loss to follow-up was addressed | Not applicable (cross-sectional) |
|  | 12(e) | Describe any sensitivity analyses | None pre-specified |
| **Results** |  |  |  |
| Participants | 13(a) | Report numbers of individuals at each stage of study—eg numbers potentially eligible, examined for eligibility, confirmed eligible, included in the study, completing follow-up, and analysed | Figure 1A; §3.1 |
|  | 13(b) | Give reasons for non-participation at each stage | Figure 1A |
|  | 13(c) | Consider use of a flow diagram | Figure 1A |
| Descriptive data | 14(a) | Give characteristics of study participants (eg demographic, clinical, social) and information on exposures and potential confounders | Table 1; §3.1 |
|  | 14(b) | Indicate number of participants with missing data for each variable of interest | §2.2 (n = 4 excluded) |
| Outcome data | 15 | Report numbers of outcome events or summary measures over time | Tables 1, 2; §3.1-3.4 |
| Main results | 16(a) | Give unadjusted estimates and, if applicable, confounder-adjusted estimates and their precision (eg 95% confidence interval) | Tables 1, 2; Figures 1-2 |
|  | 16(b) | Report category boundaries when continuous variables were categorized | §2.5 (H-Y stage groups) |
| Other analyses | 17 | Report other analyses done—eg analyses of subgroups and interactions, and sensitivity analyses | §3.4 (intra-stage heterogeneity) |
| **Discussion** |  |  |  |
| Key results | 18 | Summarise key results with reference to study objectives | §4 (p.10) |
| Limitations | 19 | Discuss limitations of the study, taking into account sources of potential bias or imprecision | §4.3 (p.12) |
| Interpretation | 20 | Give a cautious overall interpretation of results considering objectives, limitations, multiplicity of analyses, results from similar studies, and other relevant evidence | §4, §4.2 (pp.10-12) |
| Generalisability | 21 | Discuss the generalisability (external validity) of the study results | §4.3 (p.12) |
| **Other information** |  |  |  |
| Funding | 22 | Give the source of funding and the role of the funders for the present study and, if applicable, for the original study on which the present article is based | Declarations (p.13) |

*Reference: von Elm E, Altman DG, Egger M, Pocock SJ, Gøtzsche PC, Vandenbroucke JP; STROBE Initiative. The Strengthening the Reporting of Observational Studies in Epidemiology (STROBE) statement: guidelines for reporting observational studies. Lancet. 2007;370(9596):1453–1457. doi:10.1016/S0140-6736(07)61602-X.*
